# Estimating how contouring differences affect normal tissue complication probability modelling

**DOI:** 10.1016/j.phro.2024.100533

**Published:** 2024-01-04

**Authors:** Miguel Garrett Fernandes, Johan Bussink, Robin Wijsman, Barbara Stam, René Monshouwer

**Affiliations:** a Department of Radiation Oncology, Radboud University Medical Center, Nijmegen, The Netherlands; b Department of Radiation Oncology, University Medical Center Groningen, Groningen, The Netherlands; c Department of Radiation Oncology, The Netherlands Cancer Institute, Amsterdam, The Netherlands

**Keywords:** NTCP, Automatic contouring, Monte Carlo, Radiotoxicity, Heart, NSCLC

## Abstract

**Background and purpose:**

Normal tissue complication probability (NTCP) models are developed from large retrospective datasets where automatic contouring is often used to contour the organs at risk. This study proposes a methodology to estimate how discrepancies between two sets of contours are reflected on NTCP model performance. We apply this methodology to heart contours within a dataset of non-small cell lung cancer (NSCLC) patients.

**Materials and methods:**

One of the contour sets is designated the ground truth and a dosimetric parameter derived from it is used to simulate outcomes via a predefined NTCP relationship. For each simulated outcome, the selected dosimetric parameters associated with each contour set are individually used to fit a toxicity model and their performance is compared. Our dataset comprised 605 stage IIA-IIIB NSCLC patients. Manual, deep learning, and atlas-based heart contours were available.

**Results:**

How contour differences were reflected in NTCP model performance depended on the slope of the predefined model, the dosimetric parameter utilized, and the size of the cohort. The impact of contour differences on NTCP model performance increased with steeper NTCP curves. In our dataset, parameters on the low range of the dose-volume histogram were more robust to contour differences.

**Conclusions:**

Our methodology can be used to estimate whether a given contouring model is fit for NTCP model development. For the heart in comparable datasets, average Dice should be at least as high as between our manual and deep learning contours for shallow NTCP relationships (88.5 ± 4.5 %) and higher for steep relationships.

## Highlights

• Contours used for toxicity model development are often automatically generated.
• We propose a methodology to evaluate how contour differences impact toxicity models.
• The impact of heart contour differences on toxicity models is studied in lung cancer.
• Impact is dependent on model slope, used dosimetric parameter and dataset size.

## 1 Introduction

Automatically generated contours have increasingly been integrated into the radiotherapy workflow [1], [2]. One emerging application of automatic contouring involves the extraction of dosimetric parameters for survival analysis and the development of normal tissue complication probability (NTCP) models using large databases of retrospective radiotherapy data [3], [4]. The advantage of using automatic contouring for this purpose lies in the ability to analyze large cohorts, especially when the organs of interest are not contoured in the original dataset. Moreover, the automated nature of this contouring can enhance data consistency by reducing interobserver variability and/or differences between contouring protocols, which can pose challenges in large, multicentric datasets [5].

NTCP models establish a sigmoidal relationship between one or more dosimetric parameters and the probability of a specific outcome occurring. Developing these toxicity models from radiotherapy plans involves multiple steps [6], and the influence of contour differences on the derived models is mediated by the dosimetric parameter computed from those contours. The location of the contour differences and the shape of the dose distribution determine the impact on the computed dosimetric parameter used in the NTCP model.

While the effect of contouring differences on NTCP curves has been reported for specific contouring and survival sets [7], [8], [9], [10], comprehensive studies on this topic are lacking. Mövik et al. [11] investigated how normally distributed random errors added to mean heart dose (MHD) and mean lung dose affect NTCP curves. They observed an underestimation of NTCP values associated with the errors, as well as a decrease in NTCP uncertainty with increasing dataset size. However, contouring differences may result in dosimetric parameter variations that are not adequately captured by this type of approaches. Instead, automatic contouring models and experts often commit systematic contouring errors. Currently, it remains uncertain how accurate contouring needs to be for the purpose of developing NTCP models and what potential impact contouring inaccuracies, as well as intra and interobserver variability, have on the accuracy and generalizability of these NTCP models. Furthermore, there is a lack of research investigating the specific factors and their respective roles in mediating the translation of contour differences into differences in the derived NTCP models.

Given two sets of contours of the same organ at risk, we propose a methodology to evaluate the impact the discrepancies between the two sets of contours have on the performance of the derived NTCP models. We apply this methodology to a cohort of non-small cell lung cancer (NSCLC) patients, where manual, atlas, and deep learning (DL)-based contours of the heart were available. By doing this, we aim to show an example of how contour similarity, NTCP parameters, the selected dosimetric parameters, and dataset size influence the difference between the performance of the resulting NTCP models.

## 2 Materials and methods

### 2.1 Proposed framework

Our methodology for investigating the impact of differences between two sets of contours on toxicity modelling is schematically as follows: one set is designated as the ground truth contour set and the other as the alternative contour set. The ground truth contour set is utilized to simulate outcome data based on a predefined NTCP relationship via a given dosimetric parameter. Subsequently, the two sets of contours and the simulated outcomes are then used to fit one NTCP model each and their performances are compared. For any given predefined NTCP relationship, multiple sets of outcomes, or cohorts, can be drawn by converting the NTCP values into binary outcome labels for each patient. The statistics involved can be estimated by a Monte Carlo simulation with sufficient iterations.

**Fig. 1:**
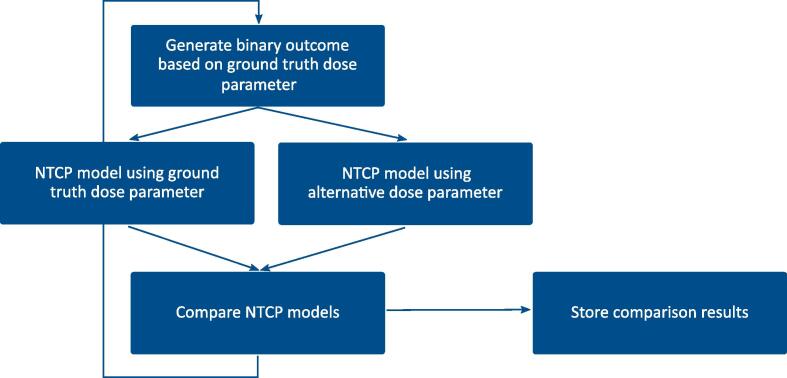
Monte Carlo iteration scheme for the analysis of the influence of contouring errors on toxicity modelling. In a Monte Carlo simulation, a dosimetric parameter is chosen and the NTCP of each patient is computed using the predefined NTCP model. In every iteration of the Monte Carlo simulation, the previously computed patient NTCPs are randomly converted to a binary outcome. The binary outcome is then used to fit a ground truth-based NTCP model and an alternative-based NTCP model. The performance of these two NTCP models is then compared, and the results are stored.

In each Monte Carlo iteration, NTCP parameters are estimated for both models and their performance compared. It is recorded whether the model derived from the ground truth contours has significantly better performance than the one derived from the alternative contour set. Statistical significance can be evaluated via bootstrapping. Fig. 1 illustrates the workflow of each Monte Carlo iteration. Additional detail on the Monte Carlo procedure is given in the Supplementary Material section S.1. Next, we present how this methodology was applied to study the impact of heart contour differences on NTCP modelling in a lung cancer dataset.

### 2.2 Data

Treatment plans and clinical contours of 605 NSCLC patients stages IIA-IIIB who were prescribed a dose of 66 Gy in 24 fractions combined with chemotherapy at the Netherlands Cancer Institute were retrospectively collected [4]. The available heart contours, created by radiation oncologists for treatment planning, were obtained for all patients. In addition to these manual contours, two other sets of contours were obtained using automatic contouring algorithms. One set was generated using a multi-atlas based non-rigid registration method [4], [12], while the other set was computed using an extensively validated DL model [13]. The manual contours did not follow consistent contouring guidelines, the atlas and DL contours followed [14]. Dice and surface Dice [19] were used to compare the agreement between contour sets. MHD and heart VxGy parameters (percentage of heart volume receiving at least x Gy) were computed for all three contour sets (V5Gy, V10Gy, and V30Gy). These dosimetric parameters have been associated with survival and cardiac complications in comparable datasets [4], [15], [16]. The usage of the data was approved by the Institutional Review Board at the NKI. The confidentiality and anonymity of the patients’ data was strictly maintained throughout the study in compliance with local regulations.

### 2.3 Applying the framework

The aforementioned framework was applied to our NSCLC dataset to study how the discrepancies between the three heart contour sets – manual, DL, and atlas – affect NTCP modelling. The NTCP model used was a logistic function as defined in Moiseenko et al. [17]:

$$\mathrm{NTCP}\left(D\right)=\frac{1}{1+e^{s\left(D_{50}-D\right)}}$$

Where D is the dosimetric parameter and

$$s=4\frac{\gamma }{D_{50}}$$

Is the model slope, where γ is the normalized slope of the NTCP curve at the $D_{50}$ point, that is:

$$\gamma =D_{50}\frac{\mathrm{dNTCP}}{\mathrm{dD}}$$

For ease of interpretation, and without loss of generality, the mean of the ground truth dose parameter, $D^{\mathrm{GT}}$, was normalized to 1 and the same transformation was applied to the dose parameters of the alternative contours. For the predefined NTCP relationship a $D_{50}=1=mean\left(D^{\mathrm{GT}}\right)$ was always chosen. Monte Carlo simulations were executed for various predefined dosimetric parameters (MHD, V5Gy, V10Gy, and V30Gy), γ values (0.1 to 1.5 in 0.1 intervals), and cohort sizes (605, 302, and 182 patients). Each simulation comprised 3000 iterations. NTCP model fitting was conducted via likelihood maximization with respect to $D_{50}$ and γ. Smaller cohort sizes were generated by randomly sampling without replacement from the full cohort in each iteration. The quality of the fit for both models was evaluated using the area under the curve (AUC) and compared for statistical significance via bootstrapping (α = 5 %). That is, in each Monte Carlo iteration, the distribution of the pairwise AUC difference between the ground truth derived model and the alternative model was estimated by conducting 100 bootstrap draws. If one model exhibited a higher AUC in over 95 % of the bootstrap draws, it was deemed statistically superior for that specific Monte Carlo iteration. Results of simulations using the manual contours as the ground truth and the DL and atlas contours as the alternative are presented here. The corresponding results using the other contour sets as the ground truth can be found in the supplementary material (Figs. S1–S6).

The Monte Carlo simulations were implemented in Python version 3.9.16, and the code is publicly accessible on GitHub [18]. A typical execution of 3000 iterations takes approximately 3 min on a 3.40 GHz Intel(R) Core(TM) i7-6700 CPU.

## 3 Results

Table 1 presents the geometric correspondence between the various contour sets in our dataset. The Dice and surface Dice values clearly indicate a higher agreement between the manual and DL contours compared to the manual and atlas contours. This agreement is also reflected by the correlation between the dosimetric parameters (Table S1), where the manual and DL-derived dosimetric parameters exhibit a stronger correlation than the manual and atlas parameters.

**Table 1:** Average Dice and Surface Dice at 3 mm tolerance between the manual contours and the DL and atlas contours.

	Manual vs DL	Manual vs Atlas
Dice	88.5 ± 4.5 %	82.2 ± 4.6 %
Surface Dice at 3 mm	67.3 ± 13.4 %	60.0 ± 7.7 %

**Fig. 2:**
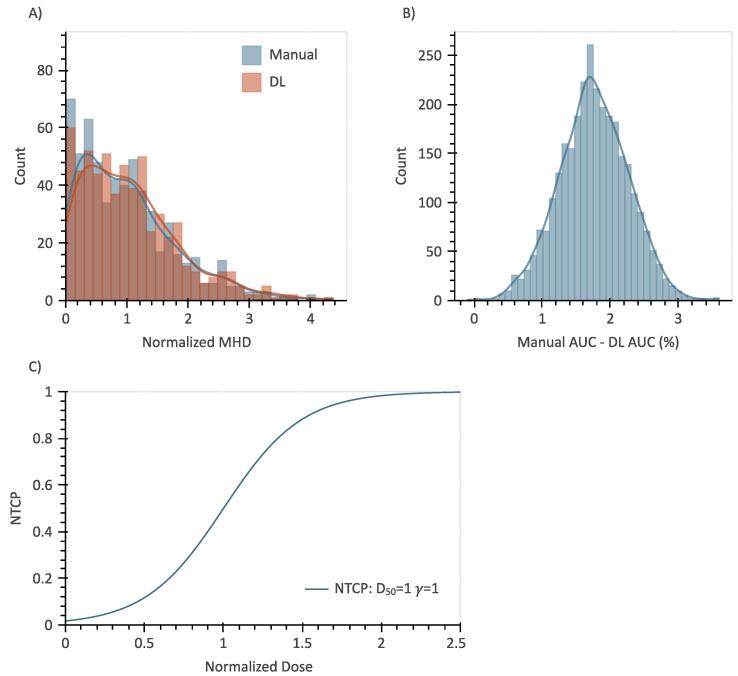
Result of a Monte Carlo simulation with an imposed toxicity model with parameters *D*_50_ = 1 and *γ* = 1. A) distribution of the normalized mean heart dose (MHD) computed from manual (blue) and DL (orange) contours. B) distribution of pairwise AUC differences for all iterations. Mean AUC difference was 1.8 % ± 0.5 %. C) shape of the NTCP curve with parameters *D*_50_ = 1 and *γ* = 1. In 94.3 % of the iterations, the AUC of the models derived from the manual contours was significantly better.

Fig. 2 illustrates the results of a single Monte Carlo simulation comparing the manual (ground truth) to the DL contours with an NTCP model with parameters $D_{50}=1$ and γ=1 and MHD as the dosimetric parameter. Fig. 2A displays the histograms of the MHD derived from the manual and DL contours, while Fig. 2B depicts the distribution of the pairwise AUC difference between the resulting 3000 manual and DL-derived models. The mean AUC difference was 1.8 ± 0.5 % (higher for ground truth derived models on average). Out of the 3000 iterations, the AUC of the models derived from the manual contours was significantly better than those derived from the DL contours in 94.3 % of the cases.

**Fig. 3:**
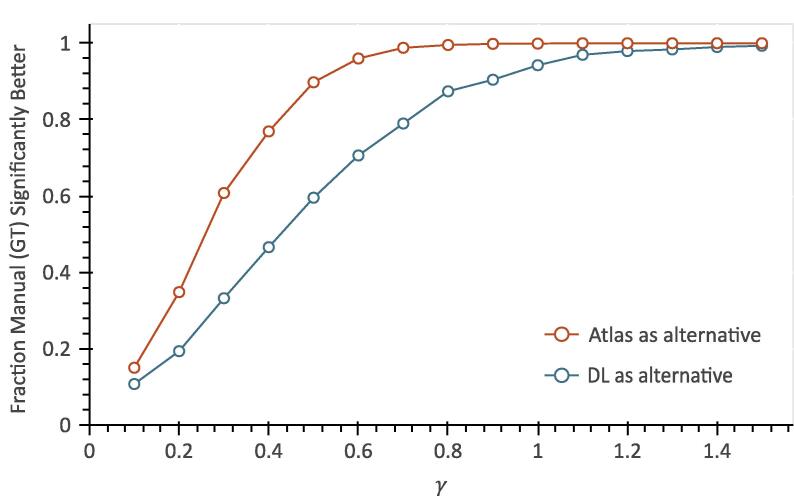
Plot of the fraction of Monte Carlo iterations where the models based on the mean heart dose (MHD) derived from the manual contours (ground truth) had significantly better AUC than those based on the MHD derived from the alternative contours. GT: Ground Truth.

Fig. 3 shows the relationship between γ, the slope of the NTCP model at $D_{50}$, and the fraction of significantly better manual contour (ground truth) derived models when MHD was used as the dosimetric parameter. With increasing γ, the AUC of the models increased, and the fraction of cohorts where the models derived from the manual contours were significantly better than those derived from the DL and atlas contours also increased. Furthermore, for any given γ value, the models derived from the manual contours were significantly superior to the models derived from the atlas contours more frequently than to those derived from the DL contours.

**Fig. 4:**
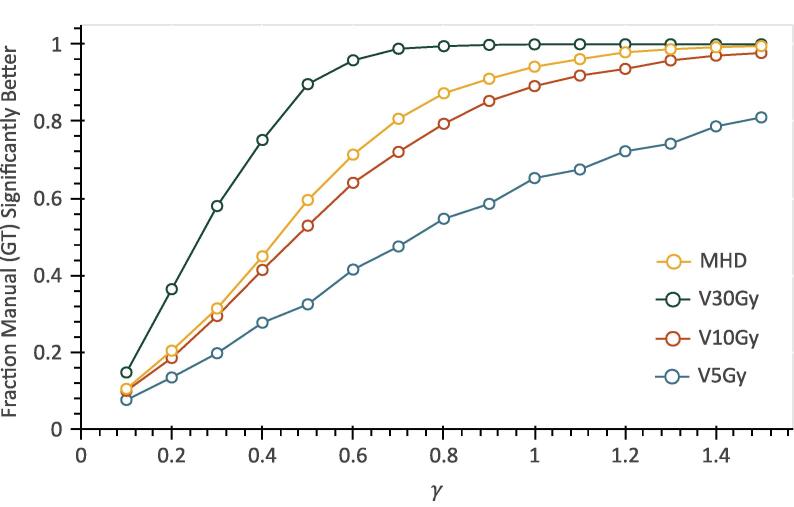
Plot of the fraction of Monte Carlo iterations where the models derived from the manual contours (ground truth) had significantly better AUC than those based on the dosimetric parameters derived from the DL contours (alternative) with respect to γ. This is shown for dosimetric parameters V5Gy, V10Gy, V30Gy, and MHD.

We also observed that the fraction of significantly better models derived from the ground truth depended on the dosimetric parameter used to simulate the outcome, as shown in Fig. 4, for the case in which the DL contour set was the alternative. The correlation between the manual and DL-derived VxGy parameters decreased with increasing x, which reflected in models with greater difference, in terms of AUC, with increasing x too. This was particularly true for higher values of γ.

**Fig. 5:**
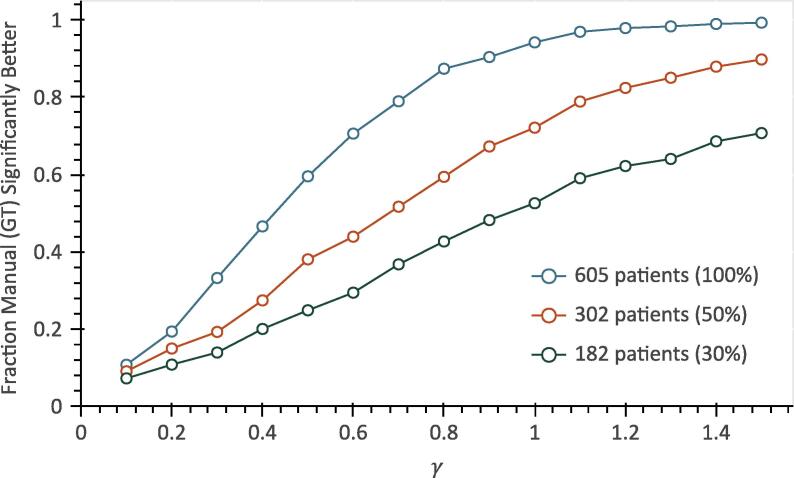
Plot of the fraction of Monte Carlo iterations where the models based on MHD derived from the manual contours (ground truth) had significantly better AUC than those based on MHD derived from the DL contours with respect to γ. This is shown for three different cohort sizes (605, 302, and 182 patients).

The cohort size also influenced the fraction of significantly better models derived from the ground truth. Fig. 5 illustrates the results for cohorts of sizes 182, 302, and 605 patients using MHD as the dosimetric parameter. Larger cohorts demonstrated a higher fraction of cases where using the manual contours led to significantly better models. The fraction of events was kept approximately the same on average for all cohort sizes. With regards to AUC difference, while the mean difference was approximately the same for all cohort sizes for any given γ value, the standard deviation decreased with increasing cohort size.

For all Monte Carlo simulations, the percentage of events was 50.0 ± 2.0 % on average for γ = 0.1 and decreased to approximately 43.6 ± 1.1 % on average for γ = 1.5 when using the full cohort and identical for the smaller cohort sizes.

## 4 Discussion

In this study, we presented a method for analyzing how discrepancies between contours impact NTCP modelling. This method consists of hypothesizing an NTCP relationship based on a ground truth contour set and toxicity model, and then testing whether the NTCP model derived from the ground truth set significantly outperforms the one derived from the alternative contour set. A Monte Carlo simulation is used to derive the statistical distribution of the performance difference. This approach enables us to estimate the percentage of instances where models based on the ground truth are expected to deliver superior performance with statistical significance, compared to those derived from alternative contour sets.

We applied this methodology to a dataset of NSCLC patients where manual, atlas and DL contours of the heart were available. Cardiac radiotoxicity is currently a major research focus in thoracic cancers [16], [20], [21]. NTCP models relating cardiovascular structure dose parameters to outcomes of interest are currently being developed from large retrospective datasets in order to enable evidence-based guidelines for cardiovascular structure sparing. The relevance of automated contouring for cardiac radiotoxicity modelling makes this dataset an ideal test case.

The performance of NTCP models depends on how much of the outcome is explained by the used dosimetric parameters and by how well the shape of the chosen model reflects the true relationship between the dosimetric parameters and the outcome. By designating a contour set as the ground truth, it is ensured that the outcome is completely defined by the dosimetric parameter derived from the chosen ground truth and NTCP shape. As a result, the ground truth derived model represents the upper bound on model performance for that specific dataset and model parameters. Any differences between the performances of the ground truth and alternative derived models can then solely be attributed to the discrepancies between the ground truth and alternative contour sets and the inherent uncertainty associated with the dichotomization of the NTCP probability into a binary outcome. Our methodology, therefore, offers an optimistic (larger than expected in real scenarios) estimation of the impact contour differences have on model performance.

Given two contour sets of a dataset, the probability of the alternative contour set leading to significantly worse models is dependent on three factors: a) the slope of the NTCP curve, b) the dosimetric parameter utilized, and c) the size of the cohort.

We observed that steeper NTCP curves resulted in higher modelling sensitivity to contouring differences. This is attributable to the increased uncertainty in converting the NTCP to binary outcome labels with shallower curves, which tends to be the dominant source of model errors in such cases, leading to worse discrimination between contour sets. Different types of outcomes are associated with varying ranges of NTCP slopes, such as shallower curves for overall survival and steeper curves for organ-specific radiotoxicity [22]. In the context of cardiac toxicity modelling, we previously estimated [23] an NTCP relationship between MHD and 1-year survival with LKB parameters $D_{50}$ = 36.63 Gy and m = 1.21 Gy (translates to γ = 0.33 Gy^−1^) using the same cohort and DL-generated contours. Simulations based on these values using the same contours as the ground truth revealed that only in 9.5 % of the cases favored the DL based model over the alternative manual based models, with a mean AUC difference of 0.2 ± 0.7 %. Another example is Beukema et al. [24] who developed an NTCP model to estimate the probability of pericardial effusion grade ≥ 2 from MHD in 216 esophageal cancer patients. Their contours were automatically computed using an atlas-based algorithm. The corresponding fitted NTCP parameters were $D_{50}$ = 36.09 Gy and γ = 0.78 Gy^−1^. In our setting and with these parameters, the ground truth derived models have a 15.0 % chance of being significantly better, with the mean AUC difference being 1.3 ± 2.4 %. Both of these examples underscore that for shallow toxicity models, typical discrepancies found between different expert contours or expert contours and contours made by DL contouring models are unlikely to lead to better NTCP models. On the other hand, for very steep models, it could be worthwhile investing more resources in improving contouring accuracy. NTCP models other than the logistic regression tend to behave similarly for the range of dose values present in the dataset [17], [25].

The same contour differences can have a different impact on the derived models, depending on the dosimetric parameter utilized. In our dataset, the correlation between VxGy parameters decreased with increasing x on average (Table S1) and that reflected in the distinguishability between the derived models, as shown in Fig. 4. Therefore, our findings suggest that, for this dataset, a model based on a lower VxGy will be more robust to contour differences and should be preferred given identical performance. Our previous work confirms that this increased robustness does not necessarily compromise model performance [23]. Using dosimetric parameters that are not as affected by contouring differences could have a major beneficial impact on the robustness of the derived models.

The size of retrospective datasets typically used for developing NTCP models ranges from several tens to a few thousand patients [26], [27]. In our dataset, with cohorts of a few hundred patients, contour differences had modest impact on model accuracy even for steep NTCP curves (Fig. 5). Naturally, smaller cohorts also increase the chance of suboptimal fitting which negatively influences the generalizability of the model to new data.

In our study, we utilized AUC as a measure of NTCP model performance due to its widespread use and cohort independence. The limitations of using AUC to measure the quality of these models have been discussed in detail elsewhere [28]. These are mitigated by the substantial size of our dataset and the estimation of the upper bound performance.

The proposed methodology has multiple applications. We mention here two examples: 1) to study which organs require better automatic contouring models than those currently available. 2) to check whether an automatic contouring model is accurate enough for development of NTCP models. For instance, studies focused on developing automatic contouring algorithms possess the ground truth used for model evaluation as well as the automatic contours themselves. Hence, they are well-equipped to employ our methodology and provide additional insights about the usability of their contouring model for NTCP model development. With an analogous set up, the same methodology presented here can also be used to assess how two different dose distribution sets (for example resulting from two different treatment planning procedures) translate into differences in the derived toxicity models. This would involve designating one of the dose distribution sets the ground truth and simulating outcomes based on it in the same fashion as described here.

As mentioned before, our methodology provides optimistic estimates of the impact of contour differences on NTCP modelling. For our dataset, this optimism is compounded by the fact that the contour sets used in this study have relatively low average Dice and surface Dice values. Taking this into account and assuming comparable dose distributions as those of our dataset, we recommend for shallow models using heart contouring models with an average Dice and surface Dice at least as high as those between the manual and DL contours of our dataset (Dice: 88.5 ± 4.5 %, surface Dice at 3 mm: 67.3 ± 13.4 %), but preferably at least 90 % and 70 % on average, respectively. Inter-observer variability of heart contours has been reported above these Dice values [14], [29]. For steep NTCP models, we frequently observed significant AUC differences between the models derived from the manual and DL contours. In these cases, contouring models with higher Dice and surface Dice should be used. Future work could apply this methodology to explore the influence of contouring differences on datasets of other cancer types and organs at risk or in multivariable NTCP models.

In conclusion, our research introduced a novel method for estimating how contouring differences affect NTCP modelling and applied it to a dataset of NSCLC patients with multiple sets of heart contours. For a given dataset, how much contour differences affect the toxicity models depends on the model slope, the dosimetric parameter used, and the cohort size. For shallow dose–response relationships, contour errors made by automatic contouring algorithms and interobserver variability are unlikely to lead to significantly different models. In our dataset, lower VxGy parameters were more robust to contouring differences. Understanding the required accuracy of automated contours for NTCP modeling, as well as identifying more robust parameters against contouring differences, can help optimize resource allocation and enhance the reliability of the toxicity models.

## CRediT authorship contribution statement

**Miguel Garrett Fernandes:** Conceptualization, Methodology, Software, Validation, Formal analysis, Data curation, Writing – original draft, Writing – review & editing. **Johan Bussink:** Supervision, Funding acquisition, Writing – review & editing. **Robin Wijsman:** Supervision, Funding acquisition, Writing – review & editing. **Barbara Stam:** Conceptualization, Data curation, Writing – review & editing, Supervision, Funding acquisition. **René Monshouwer:** Conceptualization, Methodology, Software, Validation, Formal analysis, Writing – original draft, Writing – review & editing, Supervision, Funding acquisition.

## Declaration of competing interest

The authors declare that they have no known competing financial interests or personal relationships that could have appeared to influence the work reported in this paper.
