# Supplementary material for: Estimating how contouring differences affect normal tissue complication probability modelling
Source: Phys Imaging Radiat Oncol. 2024 Jan 4;29:100533. doi: 10.1016/j.phro.2024.100533 (PMC10825684; doi:10.1016/j.phro.2024.100533)
Supplement: Supplementary data 1 [file mmc1.pdf]

Supplementary Material

S.1. Monte Carlo procedure in greater detail

In our framework, each Monte Carlo simulation consists of a sufficient number of iterations that allow the estimation of the statistics associated with a given pre-established NTCP association. In our heart contour example, we chose this number to be 3000. To help understand the Monte Carlo simulation procedure we lay here the sequence of steps involved in it (for simplicity of explanation assume a logistic NTCP shape as the one described for our example): 1) one of the contour sets is taken as the ground truth. 2) the dosimetric parameter to be used in the toxicity model, D, is chosen and computed for the ground truth contour set (GT) and the alternative contour set (AT), D^{GT} and D^{AT}. 3) a given NTCP slope, γ, and D_{50} are chosen. 4) using the ground truth dosimetric parameter, D^{GT}, and the NTCP parameters γ and D_{50}, the NTCP for each patient is computed. 5) from the NTCPs computed in 4), an outcome is simulated for each patient, creating an outcome set. 6) using the dosimetric parameters D^{GT} and D^{AT} computed in 2), and the outcome set computed in 5), two toxicity models T^{GT} and T^{AT} are fit. 7) The AUCs of T^{GT} and T^{AT} are stored and compared for statistical significance *(e.g., using bootstrap)*. Steps 5) through 7) represent an iteration and are repeated as many times as needed with different randomization seeds. For each simulation, an estimate of how the contour differences between the ground truth and the alternative contour sets translate into differences between the AUCs of the derived NTCP models is thus determined for the parameter values and dosimetric parameter chosen in 2) and 3), respectively.

S.2. Correlation between dosimetric parameters

Table S1. Correlation between dosimetric parameters computed from the manual, deep learning (DL), and atlas contours.

Dosimetric Parameter		Contours		Pearson Correlation
		Manual	DL	0.970
		Manual	Atlas	0.918
MHD		DL	Atlas	0.955
	1	Manual	DL	0.992
	5	Manual	DL	0.987
	10	Manual	DL	0.979
	15	Manual	DL	0.972
	20	Manual	DL	0.967
	25	Manual	DL	0.962
	30	Manual	DL	0.956
	35	Manual	DL	0.948
	40	Manual	DL	0.938
	45	Manual	DL	0.928
	50	Manual	DL	0.915
	55	Manual	DL	0.905
	60	Manual	DL	0.892
	65	Manual	DL	0.878
	1	Manual	Atlas	0.988
	5	Manual	Atlas	0.977
	10	Manual	Atlas	0.961
	15	Manual	Atlas	0.941
	20	Manual	Atlas	0.922
	25	Manual	Atlas	0.905
	30	Manual	Atlas	0.884
	35	Manual	Atlas	0.858
	40	Manual	Atlas	0.830
	45	Manual	Atlas	0.801
	50	Manual	Atlas	0.768
	55	Manual	Atlas	0.742
	60	Manual	Atlas	0.702
	65	Manual	Atlas	0.657
	1	DL	Manual	0.992
	5	DL	Manual	0.987
	10	DL	Manual	0.979
	15	DL	Manual	0.972
	20	DL	Manual	0.967
	25	DL	Manual	0.962
	30	DL	Manual	0.956
	35	DL	Manual	0.948
	40	DL	Manual	0.938
	45	DL	Manual	0.928
	50	DL	Manual	0.915
	55	DL	Manual	0.905
	60	DL	Manual	0.892
V	65	DL	Manual	0.878

S.3. Results of a single Monte Carlo simulation

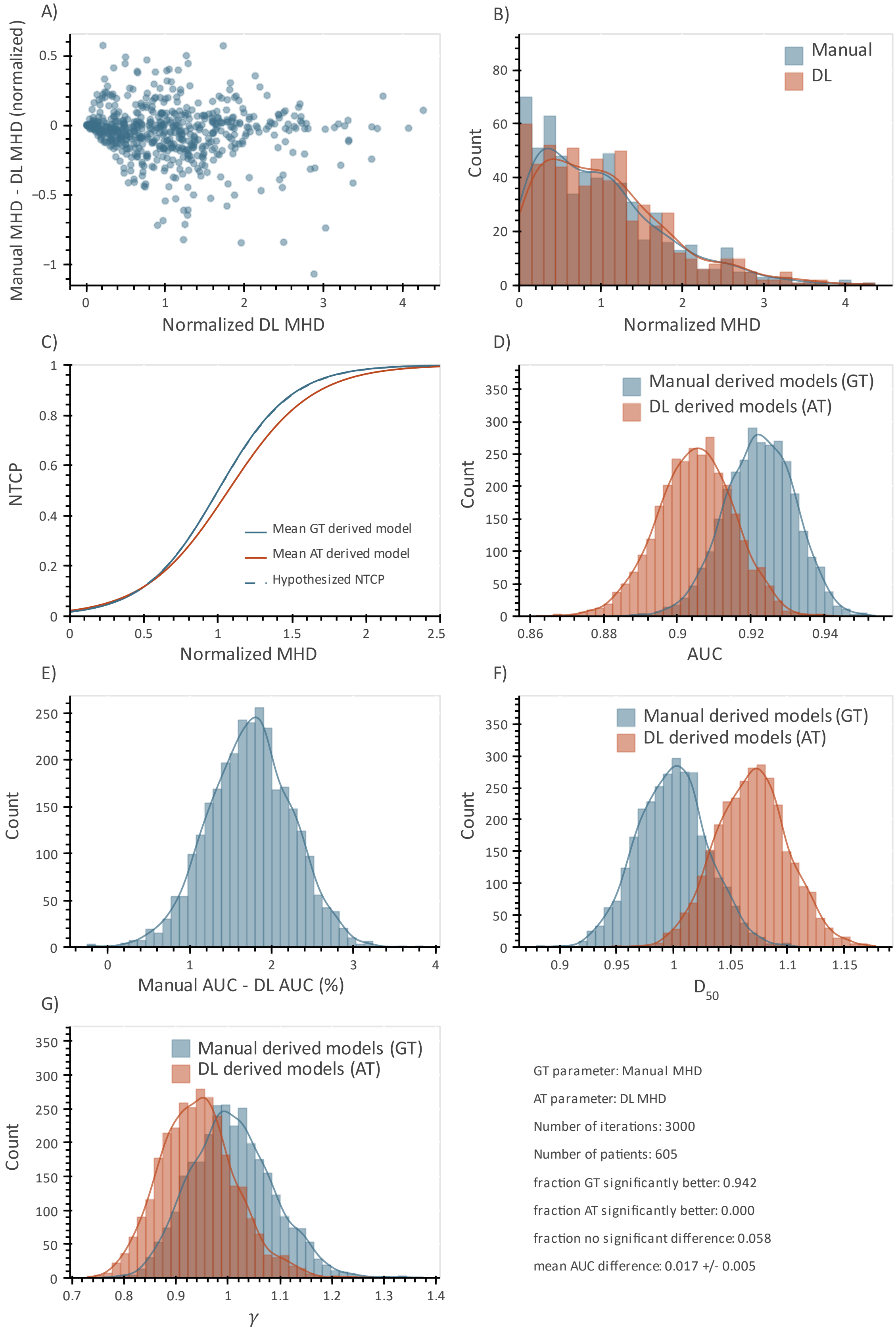

Figure S1. Results of one MC simulation using mean heart dose (MHD) as the dosimetric parameter, $\gamma = 1$ and $D_{50} = 1$ as pre-defined NTCP parameters, with manual contour as the GT and DL as the AT. GT based average model parameters: $\gamma = 1.01 \pm 0.08$, $D_{50} = 1.00 \pm 0.03$. AT based average model parameters: $\gamma = 0.94 \pm 0.07$, $D_{50} = 1.07 \pm 0.03$.

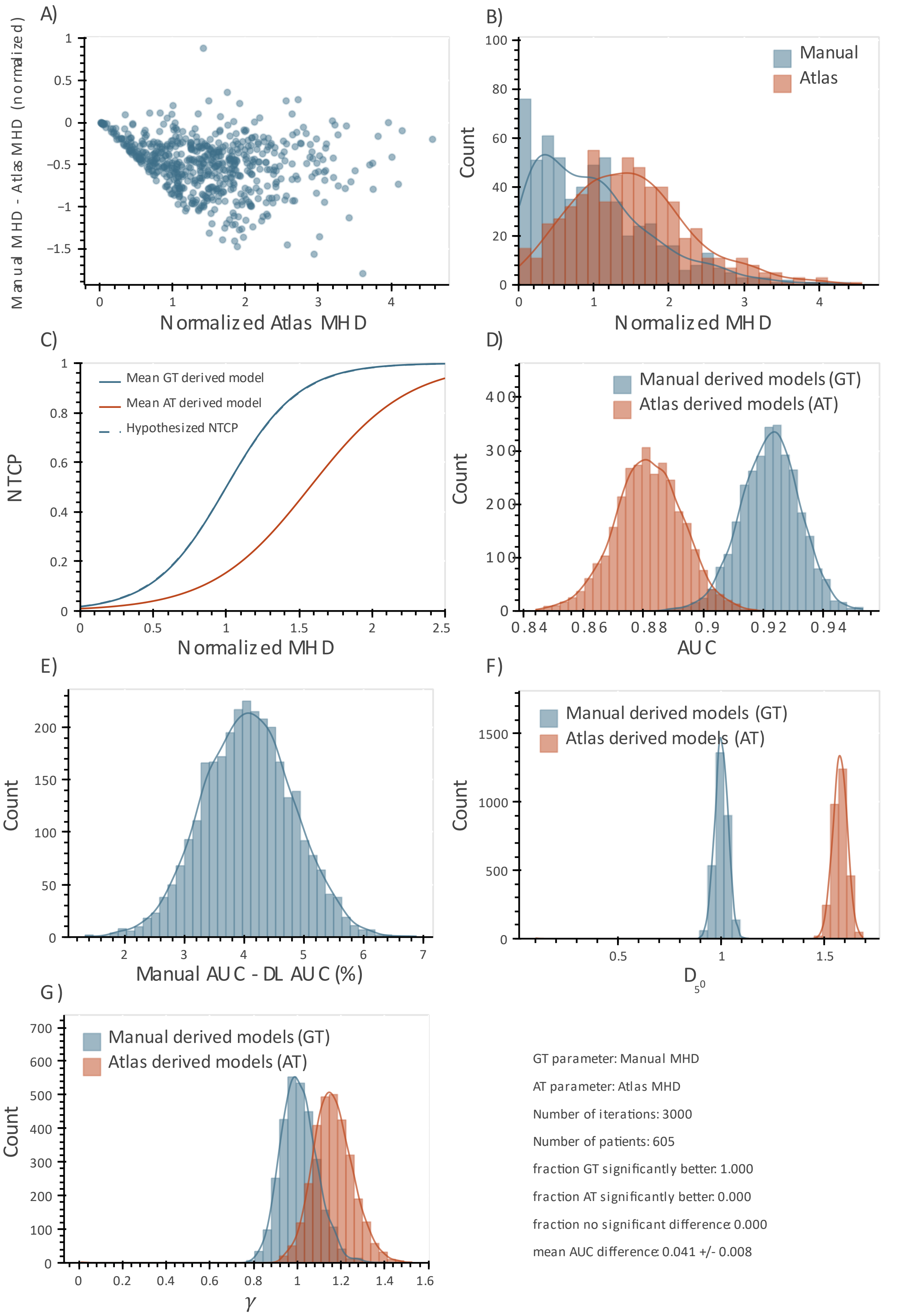

Figure S2. One MC simulation using MHD as the dosimetric parameter, $\gamma = 1$ and $D_{50} = 1$ as pre-defined NTCP parameters, with manual contour as the GT and atlas as the AT. GT based average model parameters: $\gamma = 1.01 \pm 0.08$, $D_{50} = 1.00 \pm 0.03$. AT based average model parameters: $\gamma = 1.16 \pm 0.09$, $D_{50} = 1.57 \pm 0.03$.

S.4 Additional results

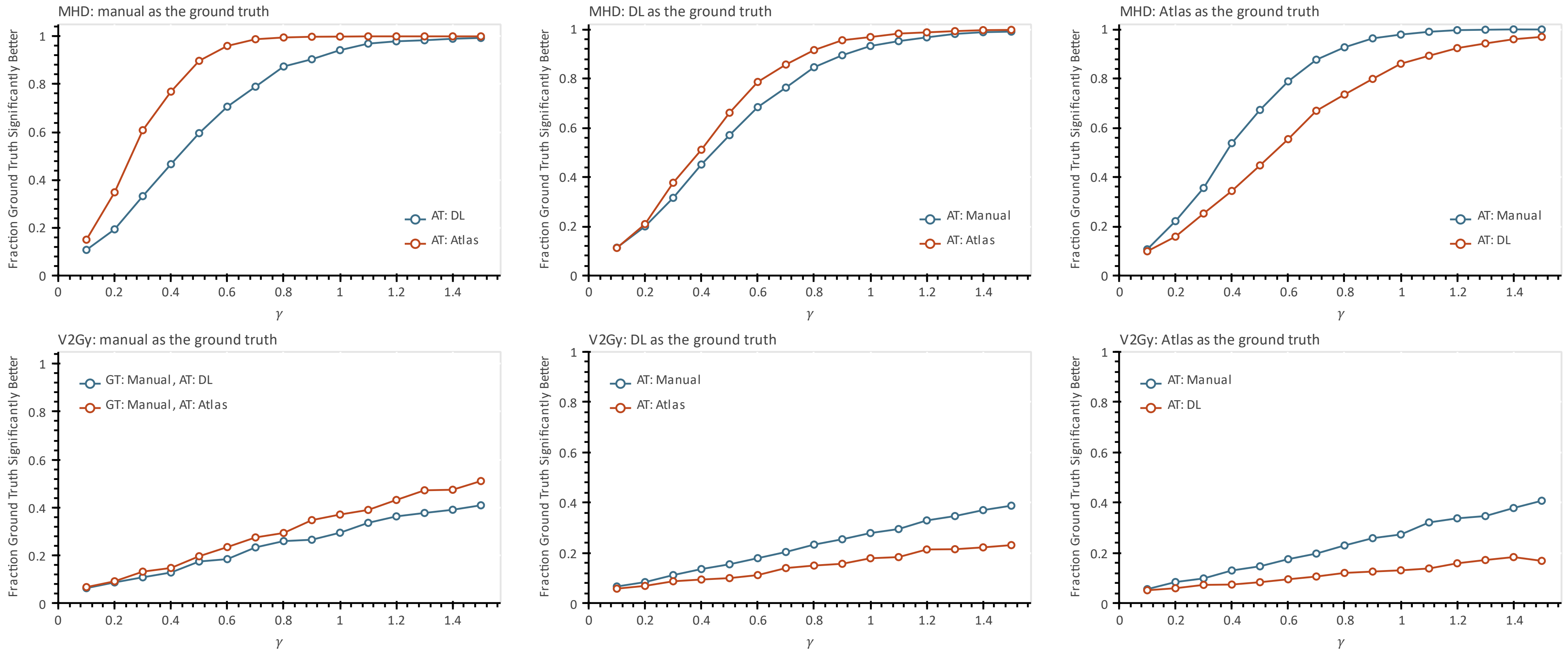

Figure S3. MHD and V2Gy. The first column shows the fraction of significantly better ground truth-derived models when the manual contours were considered the ground truth versus the predefined γ value. Results are shown for when the DL contours were considered the alternative and when the atlas contours were considered the alternative. The top row shows the results when MHD was used as the dosimetric parameter while the bottom row shows the results when heart V2Gy was used as the dosimetric parameter. The second and third columns show the corresponding results when assuming the DL and atlas contours as the ground truth respectively. The more discrepant contour sets (manual vs atlas compared to manual vs DL for instance) led to higher fractions of significantly better models derived from the ground truth. Even for very shallow predefined models there is a remarkable difference in the fraction of significantly better ground truth models between the cases when MHD was utilized as the dosimetric parameter and the cases when heart V2Gy was utilized as the dosimetric parameter. MHD is significantly more sensitive to contour differences than heart V2Gy.

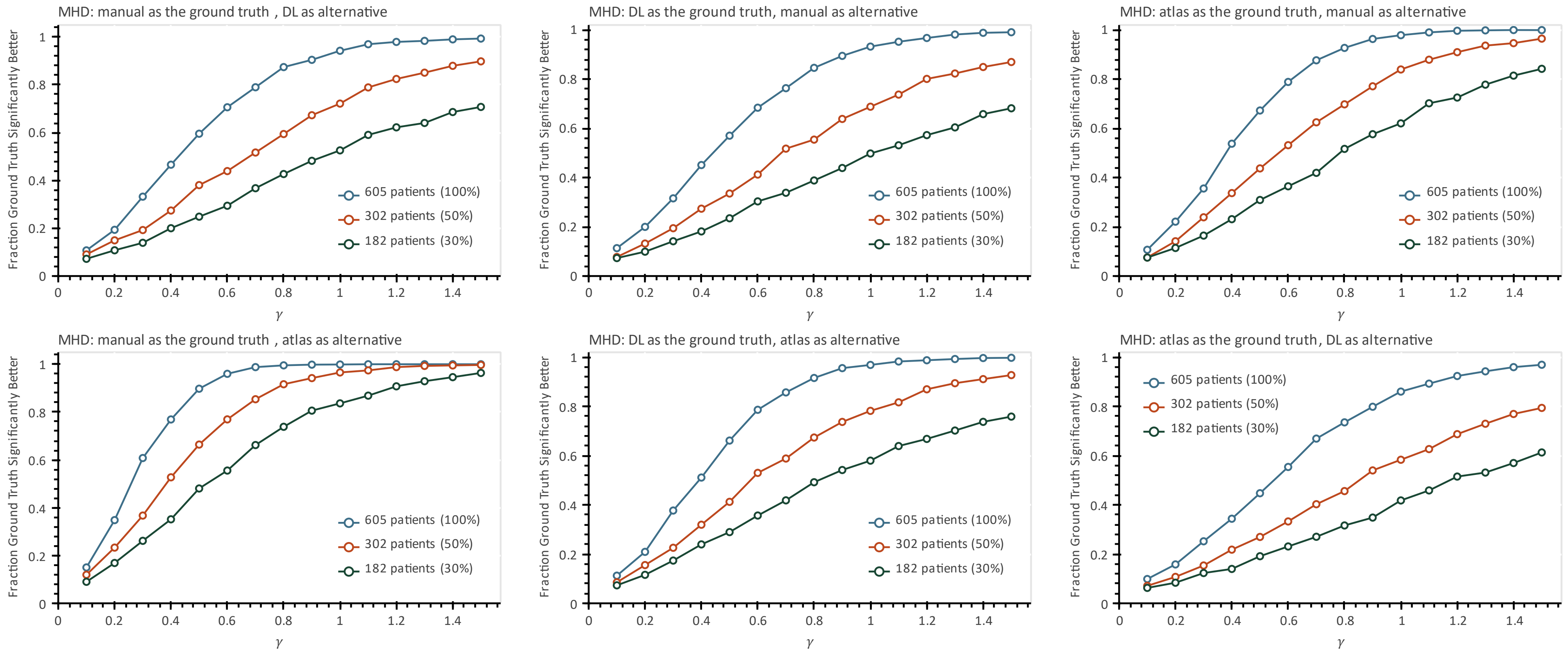

Figure S4. Cohort Size Effect. The first column shows the fraction of significantly better ground truth-derived models when the manual contours were considered the ground truth versus the predefined γ value. Results are shown for cohort sizes of 605, 302, and 182 patients. The second and third columns show the corresponding results when assuming the DL and atlas contours as the ground truth respectively. For smaller cohorts the AUC variance was larger and the AUC improvement by using the ground truth contours was smaller.

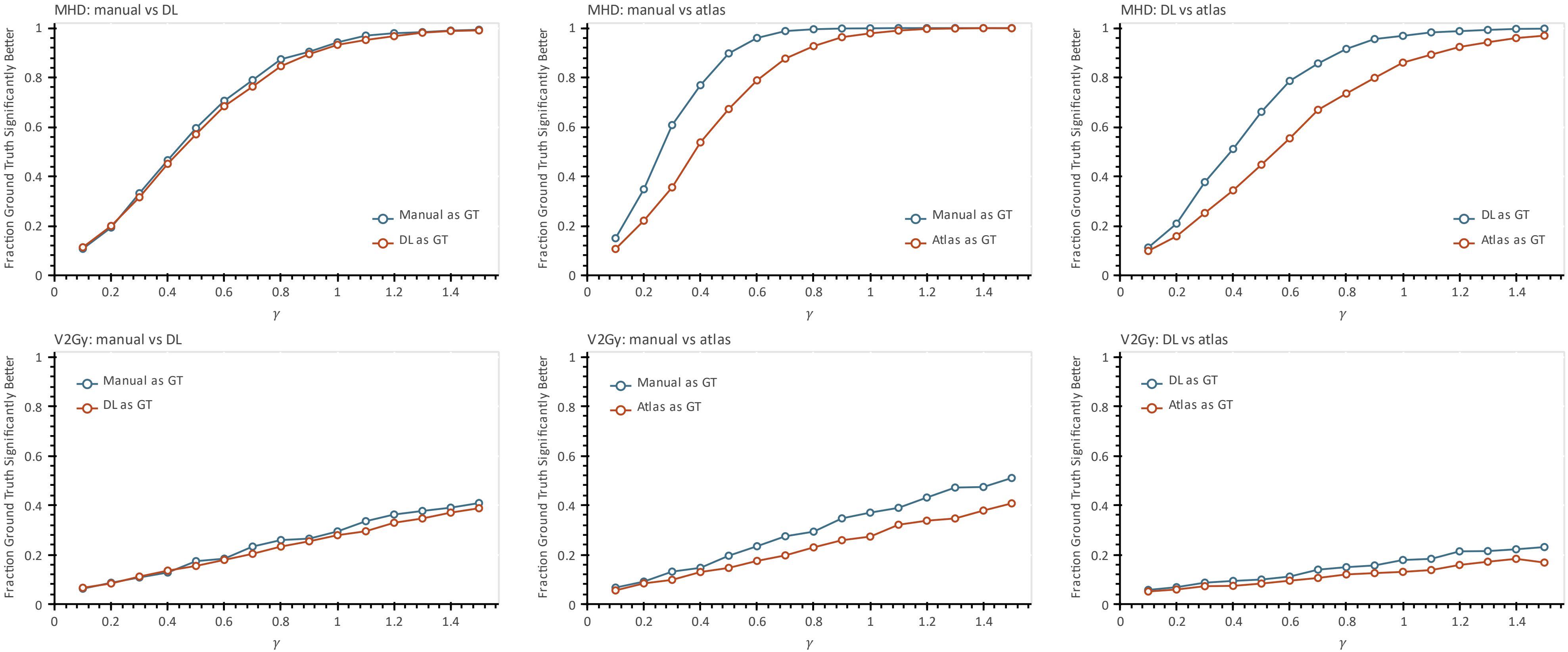

Figure S5. GT contour set effect. The first column shows the fraction of significantly better ground truth-derived models when the manual contours were considered the ground truth and the DL contours the alternative and vice-versa versus the predefined γ value. Results are shown for MHD and heart V2Gy. The second and third columns show the corresponding results when comparing manual vs atlas contours and DL vs atlas contours. The contour used as the ground truth matters for the fraction in which the models are significantly better, even though the contour sets and the dosimetric parameter values are the same. The asymmetry seems larger for more discrepant contour sets and for MHD compared to heart V2Gy.

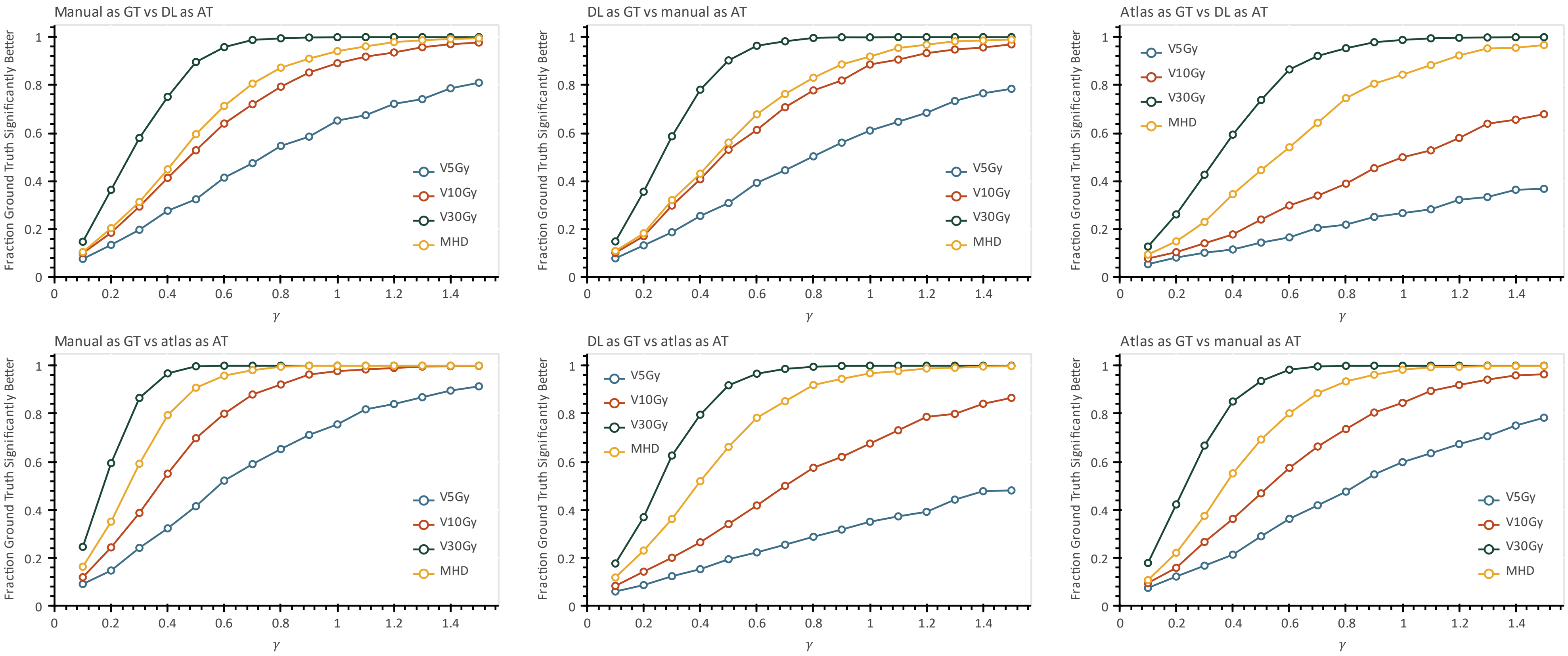

Figure S6. Effect of dosimetric parameter. The first column shows the fraction of significantly better ground truth-derived models when the manual contours were considered the ground truth versus the predefined γ value. Results are shown for heart V5Gy, V10Gy, V30Gy, and MHD. The second and third columns show the corresponding results when assuming the DL and atlas contours as the ground truth respectively. In all cases, the lower Vxs were less sensitive to contour differences for any given γ value. The correlation between the various sets of Vxs decreased with increasing x.
